# Machine learning-assisted computation of the solubility of solid drugs in supercritical carbon dioxide

**DOI:** 10.3389/fchem.2026.1883845

**Published:** 2026-07-30

**Authors:** Wenrui Ban, Bing Zhang, Chuying Wang, Yuxing Zhang, Zhiqiang Cheng, Haoyu Wang, Zheng Zhu

**Affiliations:** 1 The Second Affiliated Hospital of Xi’an Jiaotong University, Xi’an, Shaanxi, China; 2 Biomedical Research Center of Xijing University, Xi’an, Shaanxi, China; 3 Department of Orthopedics, The First People’s Hospital of Jinzhou District, Dalian, Liaoning, China; 4 Department of Urology, Xijing Hospital, Fourth Military Medical University, Xi’an, China

**Keywords:** artificial neural network, feature importance analysis, solid drug, solubility, supercritical carbon dioxide

## Abstract

This research applies three types of machine learning models, i.e., artificial neural network models (radial basis function, multi-layer perceptron, cascade feed-forward, and Elman recurrent neural networks), adaptive neuro-fuzzy inference systems, and least-squares support vector machines, to determine the solubility of twenty-eight solid drugs in supercritical carbon dioxide (scCO_2_). These intelligent models estimate the solubility as a function of operating conditions (equilibrium pressure and temperature), properties of solid drugs (critical temperature, critical pressure, acentric factor, molecular weight, and melting point), and scCO_2_ density. The relevancy analysis based on Pearson’s coefficient suggests that there is a meaningful relationship between the drug solubility in scCO_2_ and these input features. Simultaneously applying feature importance analysis with the hyperparameter tuning of machine learning models results in precise modeling for the considered problem. The statistical analyses supported the priority of the multi-layer perceptron (MLP) neural network over other paradigms. This model estimates the testing datasets with the average absolute relative deviation of 16.76%, the mean absolute error of 1.82, the relative absolute error of 12.29%, the root mean squared error of 3.94, and a correlation coefficient of 0.98314. This means that the MLP neural network offered the highest accuracy for predicting the solubility of the considered drugs in the supercritical solvent.

## 1 Introduction

Currently, there is a significant interest in the exploration of supercritical fluid technologies as a replacement for traditional particle formation processes in the food and pharmaceutical industries (Fages et al., 2004; Perrut et al., 2019). Compared to the conventional processes, this innovative technology presents numerous advantages, including high transfer rates, a diminished number of unit operations, lower operating costs, and environmentally friendly attributes (Hiyoshi et al., 2010; Zandifar et al., 2025). Carbon dioxide (CO_2_) stands out as the most extensively utilized supercritical fluid due to its mild critical conditions, cost-effectiveness, non-flammability, non-toxic nature, ready availability, and recyclability (Chao et al., 2025; Wang and Su, 2020).

The solubility of pharmaceutical and chemical materials in supercritical CO_2_ (scCO_2_) represents a pivotal design limitation in many supercritical applications. Consequently, the determination of solubility data stands as the most important step for both the initial feasibility assessment and the ultimate process design in the initial stages of developing any supercritical application. Due to the considerable expenses, uncertainties, complexities, and time requirements associated with experimental studies, many researchers have sought to predict this thermodynamic property using various theoretical methods. The density-based empirical models and thermodynamic equations of state-based models stand out as the most preferred choices. However, despite their extensive application, robust correlation with supercritical solubility data, and satisfactory accuracy, these models exhibit certain limitations. Apart from relying entirely on the experimental data, the effective utilization of thermodynamic models demands careful selection of appropriate equations and mixing laws, along with knowledge of the thermo-physical properties of the desired solute, which are often not readily available (Ardestani et al., 2020; Barzegar et al., 2024).

In recent times, there has been a growing adoption of machine learning in various domains of pharmaceutical science and technology (Wang et al., 2025; Yu et al., 2026). This increase is attributed to their flexibility and capability to model both linear and nonlinear systems without requiring prior knowledge of an empirical model (Jouyban et al., 2004). As a well-known machine learning method, artificial neural networks (ANNs) hold a significant advantage over traditional fitting methods and empirical correlations in certain chemical applications. Also, they have proven to be highly effective in modeling complex and multivariate dependent processes. Additionally, in certain instances, their superior prediction accuracy compared to traditional mathematical models has been substantiated (Iza et al., 2006). It is important to highlight that the application and modeling capability of the ANNs require a substantial amount of experimental data covering a comprehensive range of all variables.

Because of the nonlinear nature of solubility, the ANN method can be considered as an alternative tool for its modeling. For this reason, this technique has recently gained significant attention in the estimation and prediction of solid solubility in scCO_2_. Gharagheizi et al. employed an optimized three-layer feed-forward ANN model to calculate the solubility of 21 widely used industrial solid compounds in scCO_2_ (Gharagheizi et al., 2011). The designed ANN model presented a regression coefficient of 0.9533 and the average absolute relative deviation of 14% when compared to the experimental values. Furthermore, the ANN method has been previously validated for its exceptional accuracy in estimating and predicting the supercritical solubility of numerous pharmaceutical ingredients, including silymarin (Yang et al., 2017), Chloroquine (Ardestani et al., 2022), some anticancer drugs (Amani and Saadati, 2024), and numerous solid medicines (Abdallah el hadj et al., 2017).

The novelty of the present study lies in comparing the prediction accuracy of six machine learning paradigms, including MLP, cascade feed-forward (CFF), radial basis function (RBF), Elman recurrent (ER) neural networks, least squares support vector machine (LSSVR), and adaptive neuro-fuzzy inference system (ANFIS), using a unified database containing 822 experimental solubility measurements for 28 pharmaceutical compounds in supercritical CO_2_. Unlike previous studies that primarily focused on a single modeling approach or limited datasets, this work integrates feature relevance analysis, exhaustive hyperparameter optimization through grid search, systematic ranking of competing models, and applicability domain analysis within one unified framework. Moreover, to the best of our knowledge, this is the first study to investigate the applicability of the Elman recurrent neural network for predicting drug solubility in supercritical CO_2_. These contributions provide a more comprehensive evaluation of machine learning methodologies and offer practical guidance for selecting reliable predictive models in supercritical pharmaceutical process design.

## 2 Materials and methods

### 2.1 Data collection and characterization

This study collects and analyzes the equilibrium behavior of 28 scCO_2_-drug binary systems from the literature. Table 1 reports the drugs’ name, their distribution, and the source of the experimental data.

**TABLE 1:** The source of experimental data and their distribution per drug type.

Drug name	Number of data	Frequency (%)	References
Chloroquine	32	3.89	Ardestani et al. (2022)
Alendronate	28	3.41	Abourehab et al. (2022a)
Azathioprine	24	2.92	Sodeifian et al. (2020a)
Busulfan	32	3.89	Pishnamazi et al. (2020)
Capecitabine	40	4.87	Yamini et al. (2012)
Docetaxel	40	4.87	Yamini et al. (2012)
Cephalexin	27	3.28	Shojaee et al. (2014)
Chlorothiazide	20	2.43	Majrashi et al. (2023)
Chlorpromazine	45	5.47	Alharby et al. (2023)
Decitabine	32	3.89	Pishnamazi et al. (2021)
Dexamethasone	15	1.82	Chim et al. (2012)
Ethosuximide	32	3.89	Zha et al. (2019)
Favipiravir	28	3.41	Sajadian et al. (2022a)
Febuxostat	24	2.92	Abourehab et al. (2022b)
Fenoprofen	32	3.89	Zabihi et al. (2020)
Fludrocortisone Acetate	28	3.41	Amani et al. (2022)
Hyoscine	45	5.47	Hani et al. (2023)
Lenalidomide	28	3.41	Sajadian et al. (2022b)
Metformin	24	2.92	Venkatesan et al. (2023)
Metoprolol	28	3.41	Alshahrani et al. (2023a)
Minoxidil	24	2.92	Sodeifian et al. (2020b)
Montelukast	28	3.41	Sajadian et al. (2022c)
Sorafenib tosylate	24	2.92	Sodeifian et al. (2020c)
Tamoxifen	32	3.89	Alshahrani et al. (2023b)
Temozolomide	32	3.89	Zabihi et al. (2021)
Clozapine	27	3.28	Hosseini et al. (2010)
Lamotrigine	36	4.38	Hosseini et al. (2010)
Gambogic Acid	15	1.82	Xiang et al. (2019)

The statistical specifications (minimum, maximum, average, and standard deviation) of the involved drug characteristics and equilibrium behaviors of scCO_2_-drug systems are summarized in Table 2. This table also presents drugs’ critical temperature (T_c_), critical pressure (P_c_), acentric factor (ω), molecular weight (M), melting point (T_m_), and scCO_2_ density (ρ). Since the drug type is a categorical variable, its related characteristics (T_c_, P_c_, ω, M, and T_m_) are used to make them the quantitative feature. It is also worth noting that although the density of supercritical CO_2_ is thermodynamically determined by pressure and temperature through an appropriate equation of state, it was intentionally included as an independent input descriptor because solvent density is widely recognized as one of the most influential thermodynamic variables governing solubility in supercritical fluids (Bian et al., 2016; Faress et al., 2022; Rezaei et al., 2022). In the supercritical region, particularly near the critical point, small changes in pressure and temperature may lead to substantial and highly nonlinear variations in fluid density due to the high compressibility of CO_2_. This density variation directly affects the solvent power of supercritical CO_2_ and, consequently, the dissolution behavior of solid compounds. Therefore, density provides condensed thermodynamic information that cannot be represented adequately by pressure and temperature alone. Moreover, in practical supercritical process design and simulation, CO_2_ density is routinely calculated from reliable equations of state and is commonly used as a state variable in empirical and thermodynamic solubility correlations (Türk et al., 2010). Consequently, incorporating density as an input feature enables the machine learning models to exploit this physically meaningful descriptor of solvent strength rather than relying solely on pressure and temperature, resulting in a model that is more consistent with the thermodynamic principles governing supercritical phase behavior and solubility.

**TABLE 2:** The minimum, maximum, average, and standard deviation of the solid drugs’ solubility in scCO_2_.

Variable	No. of data	Minimum	Maximum	Average	Standard deviation
T_c_ (K)	822	791.35	1087.9	910.4554	62.4996
P_c_ (bar)	822	13.39	71.08	34.72094	16.72878
ω (−)	822	0.392	1.49	0.778108	0.252934
M (mol)	822	129.16	807.879	330.5579	158.0823
T_m_ (K)	822	332	615	446.6515	77.47072
T (K)	822	308	348	324.5572	12.18056
P (bar)	822	93.7	410	234.6359	82.22668
ρ (kg/m^3^)	822	325.6	976.43	780.6877	126.9302
S (mole frac)	822	2.0 × 10^−7^	871 × 10^−5^	44.14 × 10^−5^	134.11 × 10^−5^

### 2.2 Machine learning paradigms

Machine learning is the study of computer algorithms that allow computer programs to automatically learn and improve their performance through experience. Just as many human behaviors depend on learning and training from the time they are born and begin to understand the events around them, and the type and quality of their training and learning are effective throughout their life, machines also have the capacity to learn through experience. Machines can also learn and predict, form perceptions, and take actions. This process is in the field of machine learning, which increases the efficiency and quality of the machine and the results of modeling with the concept of deep learning.

#### 2.2.1 Least-squares support vector regression

The support vector machine (SVM) is an extraordinary framework from subsets of the machine learning methodologies (Chapelle et al., 1999). In this regression framework, prediction or classification is performed by creating nonlinear decision boundaries. The SVM methodologies can show significant flexibility in classifications and regressions with different complexities due to the nature of the feature space in which non-linear decision boundaries are created. New formulations of standard SVMs have been presented to increase their efficiency; one of the most important of them is the least squares support vector machine (Suykens et al., 2002a). This technique is completely based on statistical learning theories and uses classification and regression methods to express the least squares linear system as a loss function. The least squares structure of an SVM demands only parity limitations (Hashemkhani et al., 2015). In general, in addition to classification, LSSVR is used for linear and non-linear function problems, where in the initial weight space, the correlation between structure and property is defined using a function $y_{i}=f\left(x_{i}\right)$ that, $f\left(x_{i}\right)$ can be indicated as a linear Equation 1 or non-linear function Equation 2 in the following (Suykens et al., 2002b):

$$f\left(x\right)=\omega \varphi \left(x\right)+b$$ (1)

$$f\left(x\right)=\omega^{T}\varphi \left(x\right)+b$$ (2)

where ω is the weight vector of the linear function, and b is the corresponding coefficient.

Therefore, to solve LSSVR, it is possible to solve a system of linear or non-linear equations with efficient and scalable algorithms, such as algorithms based on conjugate gradients. LSSVRs have a good relationship with regularization networks and Gaussian processes, and use weights with different adjustment coefficients. And unlike the neural network that contains many local convergences, it is unique and global (Suykens and Vandewalle, 1999).

#### 2.2.2 Artificial neural networks

An artificial neural network that has performance like biological neural networks in the human brain is one of the versatile classes of machine learning with many applications in various fields (Abio et al., 2018; Meireles et al., 2003). The ANNs, with a function like the human brain, process information through interconnected nodes and neurons. These interconnected nodes initially receive the inputs and process them, and finally, by applying a set of weights and biases, generate and deliver the output. ANNs are appropriate to model nonlinear relationships and can be utilized without the need for analytical functions.

##### 2.2.2.1 Multi-layer perceptron neural networks

The feed-forward multi-layer perceptron typically includes an input layer, one hidden layer, and an output layer (Huang et al., 2026). This branch of ANN has been widely applied for the estimation of the solubility in scCO_2_ (Abdi-Khanghah et al., 2018; Sodeifian et al., 2016). The ANN employed in the scCO_2_ solubility study was a three-layer back-propagation network, which used the supervised training technique and was trained by minimizing the squared error of the outputs. Optimization in the neural network during the learning step is done with changes in weights and biases using gradient descent algorithms, which is expressed according to Equation 3.

$$\gamma_{j}=f\;\left(\sum_{r=1}^{N}w_{jr}x_{r}+\beta_{j}\right)$$ (3)

where $w_{jr}$ and $\beta_{j}$ are the weight and bias from the input or hidden layer, respectively. The transfer function (f), the number of layers, the number of neurons in each layer, and the training algorithm are the main hyperparameters of artificial neural networks.

##### 2.2.2.2 Cascade feed-forward neural networks

A special class of feed-forward neural networks is cascade feed-forward, in which, in the structure of these networks, each layer is directly connected with all subsequent layers (Jin et al., 2025). This issue has caused the cascade networks to have higher weight parameters than the MLP models with the same number of layers. These networks have one neuron in each layer, and their calculations are done with the same simple formula of the sum of neurons. The general performance of these networks is such that the input independent variables enter the hidden layer and move to the output layer. In the CFF model, the backpropagation training algorithm is a method of optimization applied to minimize the deviation between the experimental and predicted data via updating the biases and weights. The CFF model provides a relation between two subsequent layers and creates direct links between the input and output layers (Hagan et al., 1997).

##### 2.2.2.3 Radial basis neural networks

The radial basis function is a substantial type of supervised feed-forward neural network (Li et al., 2015). In general, the RBF neural network method consists of a three-layer topology, where the hidden layer uses radial basis functions and the output layer utilizes linear activation functions to model the processes. It is noteworthy that the quality of RBF neural network training and its performance are affected by the spread values for the radial base activation function, and the modeling results depend on it. Therefore, assigning the number of hidden neurons and the spread amount of the radial basis activation function are very important to design the RBF model (Abdi-Khanghah et al., 2018).

##### 2.2.2.4 Elman recurrent neural network

The Elman recurrent neural network introduces feedback connections to allow the model to capture temporal dynamics and sequence dependencies in data. Proposed by Jeffrey Elman in 1990 (Elman, 1990), it extends the traditional feedforward network by including a context layer that stores the previous hidden layer’s outputs and feeds them back into the network as additional inputs at the next time step (Rodriguez et al., 1999). This feedback mechanism enables the network to maintain a form of short-term memory, making it suitable for tasks involving time-series prediction, speech recognition, and sequence modeling. The Elman network’s architecture (i.e., input, hidden, context, and output layers) allows it to learn temporal patterns and relationships that standard feedforward networks cannot effectively capture. This model can investigate the nonlinear connection between variables and define the thorough distribution of a response variable contingent on independent variables.

#### 2.2.3 Adaptive neuro-fuzzy inference systems

The adaptive neuro-fuzzy inference system is a combined approach of fuzzy logic and artificial neural network that has the advantages of both neural network and fuzzy logic (Taghavifar et al., 2015). In the overall structure, ANFIS connects the introductory and concluding sections using a set of rules. ANFIS consists of five processing layers, which are respectively (1) fuzzy formation layer, (2) fuzzy and fuzzy rules layer, (3) membership function normalization layer, (4) deterministic part of fuzzy rules, and (5) output calculation. The parameters of the membership function of the fuzzy input are determined by the adaptive layers. Further, the adaptive parameters are applied in the fourth layer. The ANFIS parameters are regulated by minimizing the observed error between experimental and predicted results by the back-propagation method.

### 2.3 Data partitioning

To prevent data leakage, the experimental database consisting of 822 measurements for 28 pharmaceutical compounds was first divided into independent training and testing datasets using the drug-wise split strategy. Instead of randomly dividing individual samples, all experimental records belonging to a given drug were assigned exclusively to either the training or the testing dataset. Therefore, no pharmaceutical compound was present in both subsets simultaneously. This approach provides a more rigorous evaluation of the model’s ability to generalize to previously unseen compounds while preventing information leakage between the training and testing datasets.

This scenario results in 699 training samples (approximately 85%) and 123 external testing samples (approximately 15%). All model development, hyperparameter optimization, and parameter tuning were performed exclusively on the training dataset, while the testing dataset was reserved solely for the final performance evaluation. Consequently, no information from the testing dataset was used during machine learning model construction.

### 2.4 Evaluation of models’ reliability

This study uses five well-established accuracy indexes, i.e., correlation coefficient (R), root mean square error (RMSE), average absolute relative deviation (AARD%), mean absolute error (MAE), and relative absolute error percent (RAE%) to evaluate the models’ performance in the training as well as testing stages. The formula of these indices is Equations 4–8 (Jin et al., 2025; Rezaei et al., 2022):

$$R=\sqrt{1-\sum_{n=1}^{N}{\left(S_{n}^{\exp}-S_{n}^{pred}\right)}^{2}/\sum_{n=1}^{N}{\left(S_{n}^{\exp}-\bar{S}\right)}^{2}}$$ (4)

$$RMSE=\sqrt{{\sum_{n=1}^{N}\left(S_{n}^{\exp}-S_{n}^{pred}\right)}^{2}/N}$$ (5)

$$AARD\%=\left(100/N\right)\times \sum_{n}^{N}\left|S_{n}^{\exp}-S_{n}^{pred}\right|/S_{n}^{\exp}$$ (6)

$$MAE=\sum_{n=1}^{N}\left|S_{n}^{\exp}-S_{n}^{pred}\right|/N$$ (7)

$$RAE\%=100\times \sum_{n=1}^{N}\left|S_{n}^{\exp}-S_{n}^{pred}\right|/\sum_{n=1}^{N}\left|S_{n}^{\exp}-\bar{S}\right|$$ (8)

All the above-mentioned indices try to measure the deviation between experimental (S^exp^) and computed (S^pred^) values of the drug solubility in scCO_2_. The number of data points (N) as well as the average value of the experimentally measured samples ( $\bar{S}$ ) also appear in some formulations.

The ranking analysis based on these indices is also used to compare models’ reliability and identify the most accurate one.

## 3 Results and discussion

This section presents the results of the relevance test, artificial intelligence (AI) model construction, model selection, and model performance assessment.

### 3.1 Correlation matrix analysis

The relevance test is a practical method to measure the strength as well as the direction of the relationship between two variables. The Pearson correlation matrix analysis is often conducted to compute the relationship between a set of x and y variables. Equation 9 expresses the mathematical formulation of the Pearson coefficient (η) (Jin et al., 2025).

$$\eta_{x,y}=\sum_{n=1}^{N}\left(x_{n}-\bar{x}\right)\left(y_{n}-\bar{y}\right)/\left(\sqrt{\sum_{n=1}^{N}{\left(x_{n}-\bar{x}\right)}^{2}}\sqrt{\sum_{n=1}^{N}{\left(y_{n}-\bar{y}\right)}^{2}}\right)$$ (9)

This factor ranges from −1 to +1, so that the latter shows the strongest indirect relationship between x and y. On the contrary, the maximum value of η states that the strongest direct relationship exists between x and y. As much as this factor moves toward zero, the relationship between x and y becomes weaker.

Although only the Pearson coefficients between the dependent variable (S) and its influential features are important from a modeling perspective, Table 3 reports the relevance between all possible pairs of variables.

**TABLE 3:** The values of Pearson’s coefficient between all pairs of variables.

Independent variables	T	P	ρ	T_c_	P_c_	ω	M	T_m_	S
T	1	0.063	−0.505	0.026	−0.030	0.047	0.033	−0.045	−0.032
P	​	1	0.741	0.026	−0.058	0.118	0.068	−0.084	−0.184
ρ	​	​	1	0.018	−0.025	0.066	0.055	−0.009	−0.152
T_c_	​	​	​	1	−0.145	−0.139	0.414	0.208	−0.212
P_c_	​	​	​	​	1	0.119	0.059	0.361	0.229
ω	​	​	​	​	​	1	−0.185	0.179	−0.136
M	​	​	​	​	​	​	1	−0.007	−0.303
T_m_	​	​	​	​	​	​	​	1	−0.261
S	​	​	​	​	​	​	​	​	1

Table 4 reports the numerical value of Pearson coefficients between drug solubility in scCO_2_ and its related influential variables. This table also describes the physical meaning of each coefficient and sorts the relationships of drug solubility in scCO_2_ with different features based on their strength. The drug solubility in scCO_2_ decreases with all independent variables other than critical pressure. In addition, drug solubility in scCO_2_ has the maximum and minimum relevance with the drug’s molecular weight and temperature, respectively. It must be noted that in a situation where a high level of scattering is available in the experimental databank, the Pearson correlation coefficient provides ambiguous or sometimes wrong anticipations (Rezaei et al., 2022).

**TABLE 4:** The physical meaning of Pearson coefficients between drug solubility in scCO_2_ and its related features.

Solubility relationships with input variables	S-T	S-P	S-ρ	S-Tc	S-Pc	S-ω	S-M	S-Tm
η	−0.032	−0.184	−0.152	−0.212	0.229	−0.136	−0.303	−0.261
Direction	indirect	indirect	indirect	indirect	direct	indirect	indirect	indirect
Strength rank	8	5	6	4	3	7	1	2

### 3.2 Hyperparameters’ tuning of the ML paradigms

All machine learning algorithms were implemented using custom-written MATLAB R2018b code, without relying on specialized toolboxes. Before model development, all input and output variables were normalized to the interval of [0,1] to eliminate the influence of differences in variable scales and to facilitate model convergence.

Hyperparameter optimization was performed through an exhaustive full grid-search over the ranges summarized in Table 5. For the LSSVR model, both leave-one-out and k-fold cross-validation were employed during hyperparameter selection. Also, different optimization algorithms were checked to perform the training stage of the ANN-based models (MLP, CFF, RBF, and ER) and the ANFIS model. The optimal architectures identified through this procedure are summarized in Table 6.

**TABLE 5:** Hyperparameters of each machine learning type and their range of investigation in full grid search.

ML paradigm	Investigated properties	Investigated range
LSSVR	Pre-tuning technique	Leave-one-out and k-fold cross-validation
	Kernel type	Linear, quadratic, cubic, polynomial, and Gaussian
Artificial neural networks	ANN type	RBF, MLP, CFF, and ER neural networks
	Number of hidden nodes	1–20
	Activation function	Linear, tangent sigmoid, logarithmic sigmoid, and Gaussian
	Training algorithm	Twelve different algorithms
ANFIS	Fuzzification strategy	Grid partitioning (2–11 clusters)
		Fuzzy C-means (2–12 clusters)
		Subtractive clustering (cluster radius of 0.1–1)
	Optimization algorithm	Backpropagation and hybrid

**TABLE 6:** The most suitable identified hyperparameter(s) for each machine learning model.

ML paradigm	The best identified properties
LSSVR	Leave-one-out pre-tuning technique and Gaussian kernel function
RBF neural network	12 hidden nodes, Gaussian and linear activation functions, and linear programming optimization
MLP neural network	19 hidden nodes, tangent and logistic sigmoid activation functions, and Bayesian regularization optimization
CFF neural network	16 hidden nodes, Gaussian and linear activation functions, and Levenberg-Marquardt optimization
ER neural network	6 hidden nodes, logistic sigmoid and linear activation functions, and scaled conjugate gradient optimization
ANFIS	Grid partitioning, fuzzification, 8 clusters, hybrid optimization

To minimize the effect of random initialization, each machine learning configuration was trained 10 times independently, and the architecture with the lowest error was selected as the final model. Training was terminated automatically when the convergence criterion of the corresponding optimization algorithm was satisfied, including achievement of the minimum performance gradient, stabilization of the objective function, or reaching the maximum number of training iterations.

### 3.3 Determination of the best architecture of the ML paradigms

Results of conducting the statistical analyses on the performances of the topology-adjusted AI-based models in the training as well as testing steps have been introduced in Table 7. This table presents the deviation between actual and predicted values of the drug solubility in scCO_2_ in terms of five uncertainty criteria, i.e., R, RMSE, AARD%, MAE, and RAE%. An AI-based model capable of estimating the experimental drug solubility data with the highest R index value and the smallest RMSE, AARD%, MAE, and RAE% values must be chosen as the most accurate one.

**TABLE 7:** The observed AARD%, MAE, RAE%, RMSE, and R between actual and predicted values of drug solubility in scCO_2_.

ML paradigm	Stage	R	RMSE	AARD%	MAE	RAE%
RBF neural network	Training	0.39032	20.30	137.56	9.98	67.79
	Testing	0.31005	17.39	143.37	8.92	71.46
MLP neural network	Training	0.98222	3.84	6.23	1.23	8.57
	Testing	0.98314	3.94	16.76	1.82	12.29
CFF neural network	Training	0.97772	4.23	8.01	1.50	10.19
	Testing	0.97219	4.52	25.60	1.96	15.74
ER neural network	Training	0.54211	41.19	65.96	8.35	60.59
	Testing	0.74696	16.20	106.60	7.97	44.77
LSSVR	Training	0.96546	5.18	9.68	1.58	10.97
	Testing	0.97406	5.72	19.69	2.08	14.48
ANFIS	Training	0.95147	6.56	14.11	2.14	14.67
	Testing	0.95144	5.56	24.34	2.02	15.22

The results presented in Table 7 reveal considerable differences in the predictive capability of the investigated machine learning paradigms. Among them, the MLP neural network achieved the highest prediction accuracy, followed by the LSSVR, CFF, and ANFIS models. In contrast, the ER and RBF neural networks exhibited the worst performance, particularly on the testing dataset.

The superior performance of the MLP neural network indicates that feed-forward architectures are more suitable for establishing the highly nonlinear relationship between drug characteristics, equilibrium conditions, and drug solubility in supercritical CO_2_. The MLP architecture provides sufficient flexibility to approximate complex nonlinear mappings while maintaining robust generalization capability, which explains its excellent predictive performance in both the training (R = 0.98222, AARD = 6.23%) and testing (R = 0.98314, AARD = 16.76%) stages. The consistently good performance of the CFF, LSSVR, and ANFIS models further confirms that machine learning methods employing global nonlinear approximation are more appropriate than localized or recurrent architectures for predicting equilibrium drug solubility in supercritical carbon dioxide.

On the other hand, the Elman recurrent neural network demonstrated unsatisfactory predictive performance. Unlike feed-forward neural networks, the Elman architecture is primarily intended for modeling sequential or time-dependent data through recurrent feedback connections that provide short-term memory. However, the present dataset consists of independent equilibrium solubility measurements rather than temporal or sequential observations. Therefore, the recurrent memory mechanism does not provide additional useful information for the learning process. Moreover, recurrent neural networks generally require substantially larger datasets to effectively optimize their recurrent weights, whereas the available database contains only 822 experimental measurements distributed among 28 pharmaceutical compounds. These factors collectively limited the learning capability of the ER model.

Similarly, the poor performance of the RBF model can be attributed to its localized approximation mechanism, in which the prediction strongly depends on the selection of radial basis centers and spread parameters. Although an exhaustive grid-search optimization was employed to determine the optimal hyperparameters, the highly heterogeneous nature of the collected database, comprising 28 pharmaceutical compounds with diverse physicochemical characteristics, limits the capability of localized basis functions to adequately capture the complex global nonlinear relationship between the input variables and drug solubility.

### 3.4 Identifying the best ML paradigm

The deviations between models’ predictions and actual drug solubility data reported in Table 7 confirm that the MLP neural network has the maximum R index and minimum RMSE, AARD%, MAE, and RAE% indexes. Hence, the MLP neural network is considered the best model to estimate the solubility of 28 solid drugs in scCO_2_.

**FIGURE 1:**
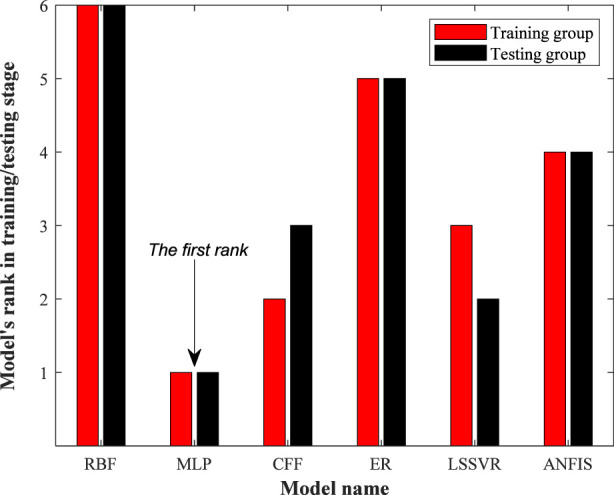
Sorting the designed machine learning models based on their performances in the training/testing stage.

The ranking test has also been conducted to systematically sort the designed models based on their performance in the training and testing steps. Indeed, the prediction performance of each machine learning model was ranked separately for the training and testing stages based on each performance metric. The final ranking for each stage was then determined by averaging the ranks obtained across all performance metrics. It should be noted that models with higher values of R and lower values of the error metrics (RMSE, AARD%, MAE, and RAE%) were assigned better ranks. Figure 1 depicts the result of such an analysis and confirms that the MLP neural network is the best model in the training as well as testing stages (i.e., the first training/testing rank). On the other hand, the RBF neural network with the worst ranking in the training/testing stage is the least accurate model for the prediction of drug solubility in scCO_2_.

### 3.5 Performance checking of the designed MLP neural network

This section relies on diverse visual inspection techniques to justify the excellent performance of the constructed MLP neural network in accurately simulating the equilibrium behavior of scCO_2_-drug binary systems.

#### 3.5.1 Visual inspection

**FIGURE 2:**
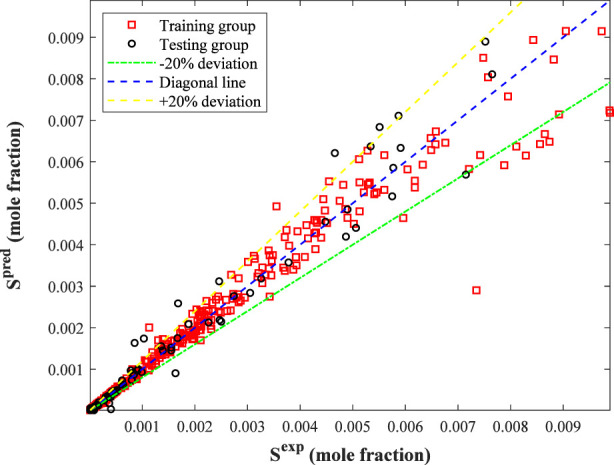
The degree of compatibility between actual and predicted drug solubilities in scCO_2_ in the training/testing stage.

Figure 2 presents the linear compatibility between experimental and computed values of drugs’ solubility in scCO_2_ by MLP neural network, separately for the training and testing stages. It can be visually concluded that the structure-tuned MLP neural network is accurate enough to simultaneously predict the solubility of 28 solid drugs in supercritical carbon dioxide.

Although this figure provides two lines showing the ±20%deviation between experimental and predicted values of the drug solubility in scCO_2_, the average absolute relative values of these deviations are 6.23% and 16.76% for the training and testing stages. Also, the observed correlation coefficients of 0.98222 and 0.98314 in the training and testing steps numerically justify the excellent compatibility between MLP neural network predictions and experimental measurements of solid drug solubility in scCO_2_.

**FIGURE 3:**
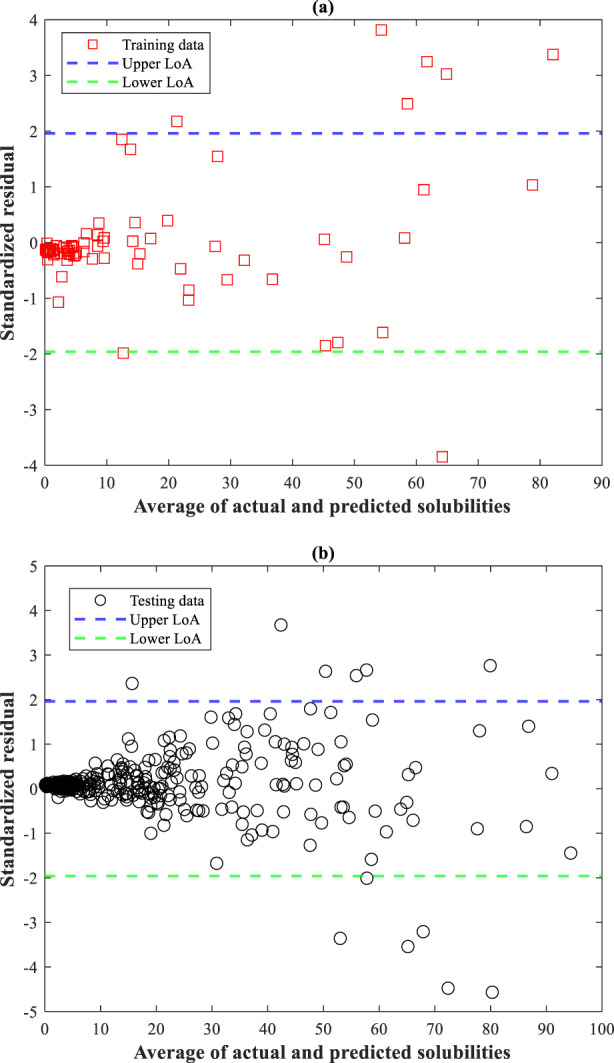
Bland-Altman plots evaluating the agreement between experimental and MLP-predicted drug solubilities for the **(a)** training and **(b)** external testing datasets.

The Bland-Altman graphs for the training and testing steps have been depicted in Figures 3a,b, respectively. This analysis plots the standardized residuals (STR) against the average values of experimental and predicted drug solubilities. For calculating the STR values, it is necessary to first compute the residual error (RE) using Equation 10.

$$RE_{n}={\left(S^{\exp}-S^{pred}\right)}_{n},n=1,2,3,...,N$$ (10)

The average values ( $\bar{RE}$ ) and standard deviation (SD) of the residual errors can be computed using Equations 11, 12.

$$\bar{RE}=\sum_{n=1}^{N}RE_{n}/N$$ (11)

$$SD=\sqrt{\sum_{n=1}^{N}{\left(RE_{n}-\bar{RE}\right)}^{2}/N}$$ (12)

Values of the STR are then possible to obtain from Equation 13.

$$STR_{n}=RE_{n}/SD\;n=1,2,...,N$$ (13)

The 95% limits of agreement (LoA) for the computed STR values have also been shown by two horizontal lines on the Bland-Altman graph. The 95% limits of agreement relative to the magnitude of the measured values are ±1.96 for both the training and testing datasets.

This analysis shows that only a small number of training and testing data points are located outside the region confined by the upper and lower LoAs. Indeed, 8 out of 699 training samples (i.e., 1.14%) and 12 out of 123 of testing samples (i.e., 9.75%) have a standard residual higher than upper LoA or smaller than lower LoA.

**FIGURE 4:**
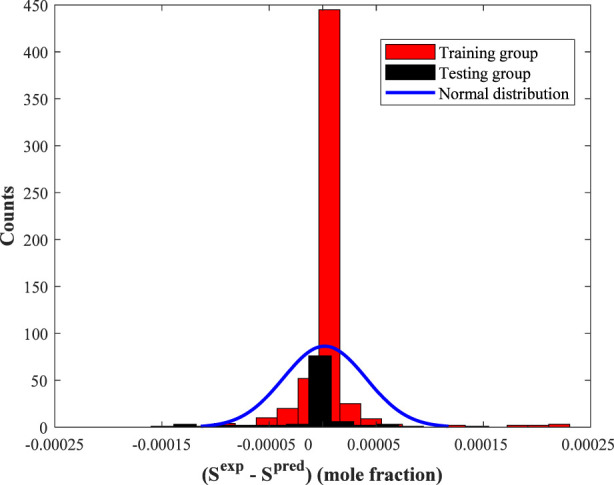
Distribution of residual errors (experimental minus predicted solubility) for the training and independent testing datasets obtained by the MLP model.

Figure 4 presents the histogram of residual errors (i.e., S^exp^ - S.^pred^) separately for the training and testing stages. This investigation reveals that almost all available experimental drug solubilities in scCO_2_ have been estimated by the MLP neural network with a residual error between −0.00005 and + 0.00005. The maximum number of training and testing samples (i.e., ∼450 and ∼80 data points) has been computed with a residual error of close to zero.

**FIGURE 5:**
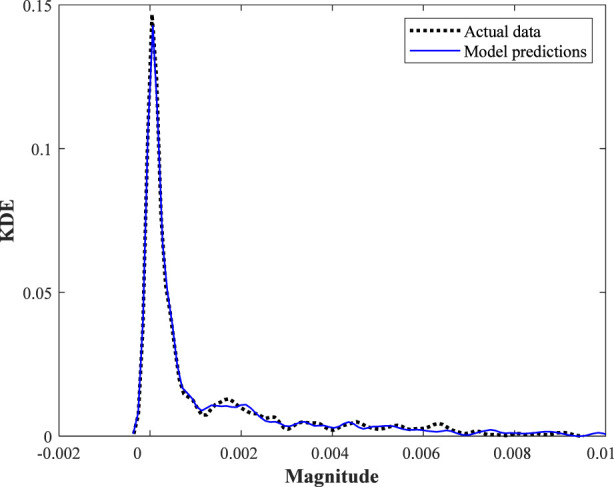
The presentation of kernel density estimation (KDE) versus magnitude of the experimental drug-scCO_2_ records and their counterpart MLP predictions.

The kernel density estimation versus the magnitude of the experimentally measured as well as estimated values of the drug solubility in scCO_2_ has been illustrated in Figure 5. This graphical analysis proves that the experimental and calculated KDE-magnitude profiles are in complete agreement and their differences are hardly observable.

#### 3.5.2 Applicability domain analysis

**FIGURE 6:**
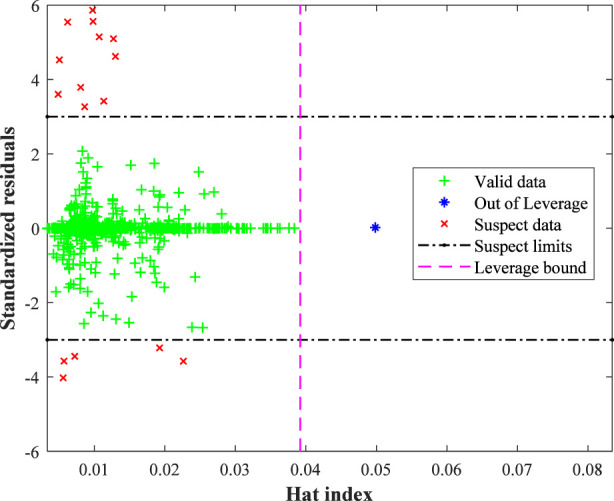
Applicability domain analysis using the leverage approach on Williams’ graph for the developed MLP model.

The applicability domain analysis is a well-established technique to detect outlier samples (suspect as well as out of leverage) amongst an experimental databank. The leverage method is a well-known method to conduct the applicability domain analysis (Faress et al., 2022). As Figure 6 presents, the leverage method plots the standardized residual against the Hat index (Equation 14) to separate valid, suspect, and out-of-leverage samples.

$$Hat\;Index=diag\left(M\;{\left(M^{t}\;M\right)}^{-1}\;M^{t}\right)\;\left\{\begin{array}{l} diagonal\;elements\;\left(diag\right)\\ transpose\;operation\left(t\right)\\ matrix\;of\;independent\;variables\;\left(M\right)\\ inversion\;operation\left(-1\right) \end{array}\right.$$ (14)

The value of the leverage boundary (Equation 15) is also required to discriminate between valid and outlier records.

$$\text{Leverageboundary}=3\times \left(\;X+1\;\right)\;/\;N,X\text{ is the number of independent variables}$$ (15)

This investigation clarifies that there are 16 suspect records and one out of the leverage sample among 822 drug-scCO_2_ equilibrium data gathered from the literature.

Table 8 reports the detailed information regarding the identified outliers and out-of-leverage data points, including the corresponding drugs, experimental conditions (temperature and pressure), and measured solubility.

**TABLE 8:** Experimental conditions corresponding to the identified outliers and out-of-leverage samples.

Drug	T (K)	P (bar)	Solubility (mole frac)	Problem	References
Favipiravir	338	120	0.000003	Suspect	Sajadian et al. (2022a)
Fenoprofen	308	400	0.000587	Suspect	Zabihi et al. (2020)
Fenoprofen	318	200	0.000355	Suspect	Zabihi et al. (2020)
Fenoprofen	318	280	0.000721	Suspect	Zabihi et al. (2020)
Fenoprofen	318	320	0.000856	Suspect	Zabihi et al. (2020)
Fenoprofen	318	360	0.000988	Suspect	Zabihi et al. (2020)
Fenoprofen	328	200	0.000466	Suspect	Zabihi et al. (2020)
Fenoprofen	328	240	0.000811	Suspect	Zabihi et al. (2020)
Fenoprofen	338	200	0.000551	Suspect	Zabihi et al. (2020)
Decitabine	328	400	0.000788	Suspect	Pishnamazi et al. (2021)
Tamoxifen	338	360	0.000829	Suspect	Alshahrani et al. (2023b)
Tamoxifen	338	400	0.000989	Suspect	Alshahrani et al. (2023b)
Chloroquine	328	160	0.000735	Suspect	Ardestani et al. (2022)
Chloroquine	338	160	0.000596	Suspect	Ardestani et al. (2022)
Chloroquine	338	400	0.000892	Suspect	Ardestani et al. (2022)
Temozolomide	328	160	0.000752	Suspect	Zabihi et al. (2021)
Temozolomide	338	120	0.000430	Out of leverage	Zabihi et al. (2021)

It must be noted that all these identified outliers and out-of-leverage points were retained in the dataset and were not excluded during the model construction. After careful evaluation, we found that the developed model maintained high predictive accuracy and robustness despite the presence of these observations. Therefore, it was concluded that the identified outliers and out-of-leverage points have a negligible impact on the overall model performance.

#### 3.5.3 Performance checking at boundary operating conditions

Tables 9, 10 provide the experimental values of the drug solubility and their associated predictions obtained by the MLP neural network at either pressure or temperature upper/lower boundaries. These tables also report the deviation between each experimental and modeling record in terms of absolute relative error (ARE%, Equation 16).

$$ARE\%=100\times \left|S^{\exp}-S^{pred}\right|/S^{\exp}$$ (16)

**TABLE 9:** Compatibility between experimental data and MLP predictions at the boundaries of the pressure.

Drug	T (K)	P (bar)	Solubility	MLP	ARE%
Montelukast	308	120	1.300 × 10^−6^	1.268 × 10^−6^	2.46
	308	150	2.400 × 10^−6^	2.212 × 10^−6^	7.84
	308	180	3.600 × 10^−6^	3.658 × 10^−6^	1.60
	308	210	4.800 × 10^−6^	5.133 × 10^−6^	6.93
	308	240	6.100 × 10^−6^	6.415 × 10^−6^	5.16
	308	270	7.400 × 10^−6^	7.575 × 10^−6^	2.36
	308	300	8.800 × 10^−6^	8.676 × 10^−6^	1.41
Docetaxel	348	182	1.390 × 10^−6^	1.313 × 10^−6^	5.57
	348	212	2.210 × 10^−6^	2.083 × 10^−6^	5.74
	348	243	3.230 × 10^−6^	3.273 × 10^−6^	1.32
	348	273	4.520 × 10^−6^	4.509 × 10^−6^	0.24
	348	304	5.310 × 10^−6^	5.676 × 10^−6^	6.90
	348	334	6.680 × 10^−6^	6.627 × 10^−6^	0.79

**TABLE 10:** Monitoring the accuracy of MLP results for the drug solubilities in the temperature boundaries.

Drug	P (bar)	T (K)	Solubility	MLP	ARE%
Gambogic acid	100	308	5.91 × 10^−6^	5.87 × 10^−6^	0.61
	100	318	2.53 × 10^−6^	2.67 × 10^−6^	5.68
	100	328	1.63 × 10^−6^	1.63 × 10^−6^	0.00
Chlorpromazine	410	318	4.21 × 10^−5^	4.02 × 10^−5^	4.50
	410	328	4.55 × 10^−5^	4.73 × 10^−5^	3.97
	410	338	4.86 × 10^−5^	5.23 × 10^−5^	7.68
	410	348	5.25 × 10^−5^	5.46 × 10^−5^	4.05

The maximum observed ARE, which is lower than 8%, approves the excellent performance of the developed MLP neural network in predicting drug solubility even at the boundaries of either pressure or temperature.

#### 3.5.4 Feature importance analysis

**FIGURE 7:**
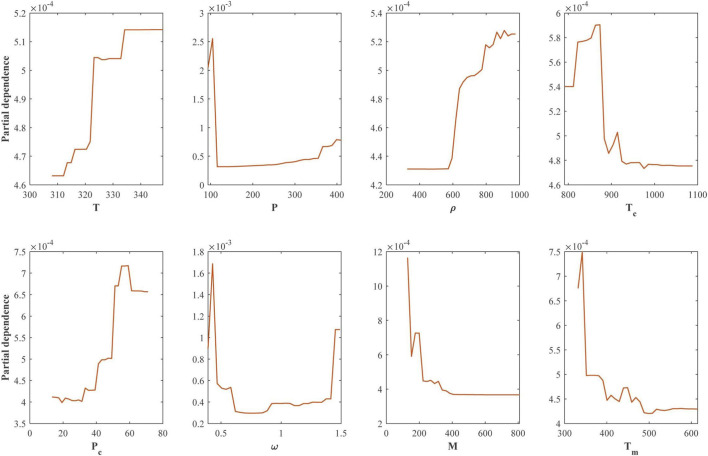
Partial dependence plots showing the influence of individual input variables on the predicted response of the MLP model.

Since solubility in scCO_2_ is highly nonlinear, Pearson correlation alone cannot adequately prove physical relevance. So, it is necessary to include other feature importance methods to strengthen such interpretations. Figure 7 presents the partial dependence plots (PDPs) of the eight input variables used in the MLP neural network to predict the solubility of drugs in scCO_2_. The PDPs reveal that the model learns predominantly nonlinear relationships, with several variables exhibiting threshold-like responses rather than simple linear trends.

This analysis indicates that increasing density consistently enhances solubility after a threshold region, reflecting the progressive increase in the solvent strength of supercritical CO_2_. Pressure exhibits a more complex response, characterized by an initial reduction followed by a gradual increase at higher pressures, suggesting that the model captures the competing influences of pressure on fluid properties and solute–solvent interactions. Temperature induces only modest changes in the predicted response, indicating that its marginal contribution is relatively weak once the effects of pressure and density are accounted for.

The acentric factor produces one of the largest variations in predicted solubility, indicating that molecular non-ideality strongly influences the dissolution behavior of pharmaceutical compounds in scCO_2_. Critical pressure displays a positive overall effect with several transition regions, whereas critical temperature has a comparatively limited and generally negative influence. Molecular weight and melting temperature both show predominantly decreasing trends, indicating that larger molecules and compounds with higher melting temperatures are predicted to possess lower solubility. These trends are physically reasonable because increasing molecular size generally reduces volatility, while higher melting temperatures are often associated with greater crystal lattice stability, both of which reduce the tendency of a compound to dissolve in a supercritical fluid.

#### 3.5.5 Comparison with the literature

This section presents both qualitative (number of data points and drugs considered) and quantitative (prediction accuracy) comparisons between the predictive models reported in the literature and the proposed MLP neural network. Table 11 summarizes the number of data points, the number of solid compounds included, and the predictive accuracy of the proposed MLP model developed in this study, alongside previously published thermodynamic equation-of-state models, empirical correlations, and machine learning approaches.

**TABLE 11:** Comparison of the generalization (number of data and drug) and predictive performance of the present study and previous studies for drug solubility in supercritical scCO_2_ (overall performance).

Checked models	Generalization^a^	The best achieved performance	References
MLP neural network	32 (1 drug)	AARD = 1.76%, R_adj_ = 0.999	Ardestani et al. (2022)
MLP neural network	128 (4 drugs)	AARD = 5.45–9.37%, R^2^ = 0.997	Amani and Saadati (2024)
ANN, LSSVR, ANFIS	254 (12 drugs)	AARD = 3.13%, R^2^ = 0.99919	Rezaei et al. (2022)
Empirical correlation	316 (12 drugs)	AARD = 9.54%	Faress et al. (2022)
ANN, equation of state, regular solution	240 (6 drugs)	AARD = 2–13%	Sodeifian et al. (2018)
Stacked machine learning	311 (12 drugs)	AARD = 8.62%, R^2^ = 0.99809	Najmi et al. (2022)
MLP, CFF, RBF, ER, LSSVR, ANFIS	822 (28 drugs)	AARD = 7.81%, R = 0.98122	This work

^a^ The number of data (number of drugs).

This investigation confirms that the proposed MLP neural network provides competitive generalization and predictive accuracy compared to those models available in the literature.

## 4 Conclusion

In this study, six machine learning models (MLP, CFF, RBF, ER neural network, LSSVR, and ANFIS) were developed and compared for predicting the solubility of 28 pharmaceutical compounds in supercritical CO_2_ using 822 experimental data points collected from the literature. All models were trained and tested using identical datasets to ensure a fair comparison. Among them, the MLP and CFF neural networks achieved the best testing performance of R = 0.98314, MAE = 1.82, RMSE = 3.94, RAE = 12.29%, and R = 0.97219, MAE = 1.96, RMSE = 4.52, RAE = 15.74%, respectively. Also, the LSSVR and ANFIS models presented acceptable accuracy for predicting the solubility of solid drugs in scCO_2_ in the testing stage, i.e., R = 0.97406, MAE = 2.08, RMSE = 5.72, RAE = 14.48%, and R = 0.95144, MAE = 2.02, RMSE = 5.56, RAE = 15.22%, respectively. Statistical and graphical validation, including residual, kernel density, Bland-Altman, applicability domain analyses, and partial dependence plot, confirmed the robustness and reliability of the best model (MLP neural network). Comparison with representative literature models further showed that the proposed MLP provides competitive generalization (number of data and number of drugs) and predictive accuracy within a unified framework applicable to a broad range of pharmaceutical compounds. The model is intended for interpolation within the investigated drugs and operating conditions, while predictions outside its applicability domain should be interpreted with caution. Future work should expand the experimental database and investigate additional machine learning and model interpretation techniques to further improve prediction accuracy and generalization.

## Data Availability

The original contributions presented in the study are included in the article/Supplementary Material, further inquiries can be directed to the corresponding authors.
